# Using Smartphones to Reduce Research Burden in a Neurodegenerative Population and Assessing Participant Adherence: A Randomized Clinical Trial and Two Observational Studies

**DOI:** 10.2196/31877

**Published:** 2022-02-04

**Authors:** Anna L Beukenhorst, Katherine M Burke, Zoe Scheier, Timothy M Miller, Sabrina Paganoni, Mackenzie Keegan, Ella Collins, Kathryn P Connaghan, Anna Tay, James Chan, James D Berry, Jukka-Pekka Onnela

**Affiliations:** 1 Department of Biostatistics Harvard T.H. Chan School of Public Health Boston, MA United States; 2 Centre for Epidemiology Versus Arthritis Manchester Academic Health Science Centre University of Manchester Manchester United Kingdom; 3 Neurological Clinical Research Institute and Sean M. Healey & AMG Center for ALS Massachusetts General Hospital Boston, MA United States; 4 Department of Neurology Washington University Saint Louis, MO United States; 5 Department of Physical Medicine and Rehabilitation Spaulding Rehabilitation Hospital Harvard Medical School Boston, MA United States; 6 MGH Institute of Health Professions Charlestown, MA United States; 7 Biostatistics Center Massachusetts General Hospital Harvard Medical School Boston, MA United States

**Keywords:** digital phenotyping, mobile health, trial, smartphones, attrition, mobile phone

## Abstract

**Background:**

Smartphone studies provide an opportunity to collect frequent data at a low burden on participants. Therefore, smartphones may enable data collection from people with progressive neurodegenerative diseases such as amyotrophic lateral sclerosis at high frequencies for a long duration. However, the progressive decline in patients’ cognitive and functional abilities could also hamper the feasibility of collecting patient-reported outcomes, audio recordings, and location data in the long term.

**Objective:**

The aim of this study is to investigate the completeness of survey data, audio recordings, and passively collected location data from 3 smartphone-based studies of people with amyotrophic lateral sclerosis.

**Methods:**

We analyzed data completeness in three studies: 2 observational cohort studies (*study 1*: N=22; duration=12 weeks and *study 2*: N=49; duration=52 weeks) and 1 clinical trial (*study 3*: N=49; duration=20 weeks). In these studies, participants were asked to complete weekly surveys; weekly audio recordings; and in the background, the app collected sensor data, including location data. For each of the three studies and each of the three data streams, we estimated time-to-discontinuation using the Kaplan–Meier method. We identified predictors of app discontinuation using Cox proportional hazards regression analysis. We quantified data completeness for both early dropouts and participants who remained engaged for longer.

**Results:**

Time-to-discontinuation was shortest in the year-long observational study and longest in the clinical trial. After 3 months in the study, most participants still completed surveys and audio recordings: 77% (17/22) in study 1, 59% (29/49) in study 2, and 96% (22/23) in study 3. After 3 months, passively collected location data were collected for 95% (21/22), 86% (42/49), and 100% (23/23) of the participants. The Cox regression did not provide evidence that demographic characteristics or disease severity at baseline were associated with attrition, although it was somewhat underpowered. The mean data completeness was the highest for passively collected location data. For most participants, data completeness declined over time; mean data completeness was typically lower in the month before participants dropped out. Moreover, data completeness was lower for people who dropped out in the first study month (very few data points) compared with participants who adhered long term (data completeness fluctuating around 75%).

**Conclusions:**

These three studies successfully collected smartphone data longitudinally from a neurodegenerative population. Despite patients’ progressive physical and cognitive decline, time-to-discontinuation was higher than in typical smartphone studies. Our study provides an important benchmark for participant engagement in a neurodegenerative population. To increase data completeness, collecting passive data (such as location data) and identifying participants who are likely to adhere during the initial phase of a study can be useful.

**Trial Registration:**

ClinicalTrials.gov NCT03168711; https://clinicaltrials.gov/ct2/show/NCT03168711

## Introduction

### Background

Participation in clinical research requires an effort. More research visits create a greater burden and, thus, a larger barrier to long-term participation. Clinical trialists often design studies that collect data relatively infrequently to reduce the burden of clinical research for participants. Although this reduces the research burden, it also reduces statistical power [1]. Data collection from participants’ smartphones may allow high-frequency data at a low burden of participation. As smartphones are now increasingly common and typically carried by their users throughout the day, every day, they can be used for nearly continuous, unobtrusive data collection in everyday settings [2,3].

This opportunity to collect research data frequently at a low participant burden is appealing for research on neurodegenerative diseases. For participants, clinic visits are especially onerous, owing to the progressive decline in their cognitive and physical function. Study teams also feel the burden on staff time, as assessment visits often are 1-3 hours in duration. Research sponsors see ballooning costs from staffing requirements [4].

Digital data collection from smartphones could reduce all of these burdens while providing relevant, quantitative, and frequent study data directly from participants. Smartphones can be used to collect a rich variety of data for clinical research. These data include *active data*, which requires data entry by the participant (eg, surveys), and *passive data* (eg, sensor and log data) that do not require activity by the participant beyond installing a research app [5]. These voluminous passive data can be converted into meaningful and interpretable variables that describe individual-level traits, habits, and behavior. If a study makes use of a participant’s own phones (therefore enabling collection of naturalistic data without requiring additional instrumentation) and collects raw high-throughput data from the phones (therefore enabling generation of study specific metrics over prepackaged metrics with enhanced reproducibility), the approach is referred to as *digital phenotyping* [6].

### Smartphone Studies in Neurodegenerative Diseases

In many cases, people with neurodegenerative diseases remain able to use their smartphones to participate in studies, despite the progressive nature of their disease. This is certainly true for people with amyotrophic lateral sclerosis (ALS), a neurodegenerative disease that causes a progressive decline in speech, respiratory function, and motor skills [7]. Recent studies have demonstrated that people with ALS use smartphones and can complete frequent surveys for research, even in the later stages of the disease [8]. Thus, smartphone-based digital phenotyping for neurodegenerative diseases is feasible.

At the same time, digital data collection has potential shortcomings that must be understood. Despite the low burden of data collection from participants’ own devices, smartphone studies may have high attrition, even when focusing on passive data collection [9-12]. When participants discontinue app use, they introduce missing active data. In addition, sensor noncollection due to technological factors or participant behavior introduces missing passive data [11,12]. Missing data, whether active or passive, reduces statistical power, threatens the generalizability of results, and can introduce attrition bias [9,12,13]. For example, if participants with more severe disease at baseline dropout more frequently, the study findings may not generalize to these participants [13].

To assess the risk of attrition bias in smartphone-based medical research, we must understand the relationship between participant characteristics, disease severity, and rate of progression on the one hand, as well as attrition risk on the other hand [9]. Patterns of attrition may differ between observational studies and clinical trials [14]. Attrition has been reported for smartphone studies in some areas, including mental health [9,15,16], cancer [17], chronic diseases [18], neurodegenerative diseases [19,20] and healthy controls [19]. However, predictors of attrition or risk of attrition bias have not been thoroughly investigated for neurodegenerative diseases. In people with diseases such as ALS, immobility, challenges with activities of daily living, and cognitive decline can threaten their ability to comply with smartphone data collection.

### Study Aims

We investigated data completeness in 3 studies using the same platform for data collection from personal smartphones of people with ALS. Two of these were observational studies, and one was a clinical trial. In all 3 studies, the participants contributed traditional ALS clinical outcome data during in-clinic visits. In addition, participants installed the front-end app from the Beiwe platform on their smartphones and used it for active and passive data collection [8]. We estimated time-to-discontinuation in each of the three studies, identified predictors of app discontinuation, and quantified data completeness for early dropouts and participants who remained engaged longitudinally.

## Methods

### Overview

We analyzed data from 3 studies using the Beiwe platform for smartphone data collection. In each of the three studies, data were collected in three ways: (1) traditional clinician-administered survey data during clinic visits or by telephone; (2) active data, including patient-reported outcomes from smartphone surveys administered through the Beiwe app, audio recordings where participants coughed, and audio recordings where participants recited a text shown on their smartphone screen; and (3) passive data from sensors and logs, automatically collected by the Beiwe smartphone app.

All data were collected and stored in compliance with local, state, and national laws, regulations, and policies. For study 1, participants were enrolled at the Massachusetts General Hospital (MGH) in Boston. For study 2, participants were enrolled at both MGH in Boston, United States, and in Washington University in St Louis, Missouri, United States. For study 3, participants were enrolled at MGH, Twin Cities ALS Clinic in Minneapolis, Minnesota, United States, and Holy Cross ALS Clinic in Fort Lauderdale, Florida, United States. The studies differed in duration and expected frequency of clinical data collection (Table 1). None of the studies included routine contact with participants to encourage engagement; there was no reimbursement for engagement; and outside of reminders from the smartphone app itself (known as notifications), no reminders were sent to participants.

**Table 1:** Characteristics of the 3 included studies.

Study	Number of participants, N	Study duration (weeks)	Frequency of data collection		
			Clinic visit	Smartphone survey	Smartphone sensors
Study 1	22	12	3 times	Weekly	GPS on for 1 minute and off for 10 minutes
Study 2	49	52	2 times	Weekly	GPS on for 1 minute and off for 10 minutes
Study 3	23	20	3 times	Weekly	GPS on for 1 minute and off for 10 minutes

### Study 1: 12-Week Pilot Study

Study 1 was a pilot observational cohort study 12 weeks in duration, running from July 2016 to June 2018. Clinician-administered survey data were collected at baseline and at weeks 6 and 12. Study design and participant recruitment for study 1 have been previously published [8].

### Study 2: 52-Week Cohort Study

Study 2 was an observational cohort study 52 weeks in duration, running from November 2018 to March 2021. Clinician-administered survey data were collected at baseline and week 52. This study used the same methods for recruitment and data collection used in study 1 [8].

### Study 3: 20-Week Clinical Trial

Study 3 was the safety of rate elevation in ALS (SURE-ALS2) randomized, placebo-controlled clinical trial of inosine to raise urate levels (NCT03168711). The trial ran from November 2017 to December 2019. The participants were divided into an intervention group, receiving inosine and a control group, receiving matching placebo until week 16. In short, after consent and successful screening for the trial, the Beiwe smartphone app was installed on the participants’ personal smartphones at the baseline visit. The app was uninstalled at the 20-week visit. Participants were asked to complete in-person visits for clinical outcomes at baseline, week 12, and week 20. They also received phone calls every 3 weeks throughout the study. The clinician-administered revised ALS functional rating scale (ALSFRS-R) was completed during in-person visits.

Study 3 had more restrictive selection criteria than the observational studies. Studies 1 and 2 required participants to have a diagnosis of ALS according to the El Escorial Criteria [21], at least moderate smartphone use, and no neurological disorders other than ALS. Study 3 included additional selection criteria requiring vital capacity >60% of predicted, plasma urate <5.5 mg/dL, and no medical history of gout, coronary artery disease, stroke, poorly controlled hypertension, or renal insufficiency.

### Ethics

Each study was approved by the Mass General Brigham Institutional Review Board (IRB). Study 2 was also approved by the Washington University IRB. Study 3 used a central IRB (the Mass General Brigham IRB) for all sites.

### Data Collection

We collected smartphone data through Beiwe, an open-source, end-to-end encrypted high-throughput digital phenotyping platform [22]. It consists of Android and iOS smartphone apps for data collection and an Amazon Web Services cloud-based system back-end for data collection and processing [23]. It has been used in both observational studies and clinical trials to collect self-administered surveys and various types of passive data [8,24].

The primary clinical outcome measure in the 3 studies was functional ability, as measured by the ALSFRS-R. The ALSFRS-R is a 12-item survey for measuring functional ability, each with 5-answer options, scored from 4 (*normal ability*) to 0 (*lowest functionality*) [25]. The questions are divided into four subdomains: the bulbar domain (questions 1-3), fine motor domain (questions 4-6), gross motor domain (questions 7-9), and respiratory domain (questions 10-12). The maximum domain score is 12 (*domain not affected*), with a lower score denoting lower functional ability. The total survey score is the sum of all questions and has a 48-point scale (from 48 points, indicating *normal function* to 0 points [25]).

### Baseline Visit

At the baseline visit, clinical characteristics were obtained in person and stored in an electronic data capture system. The Beiwe app was downloaded onto the participant’s smartphone and activated by the study coordinator. Upon activation, the app delivered a baseline survey to the participant to gather demographic and clinical information and thereafter collected active and passive data as planned.

### Smartphone Data

The Beiwe app was configured to collect weekly self-administered ALSFRS-R scores, weekly recordings of speech, and weekly recordings of cough (not analyzed here). The app also collected metadata on survey completion, including clock-times of survey presentation on the smartphone screen, submission time of each question answer by the participant, and submission time of the completed survey. In addition, the app collected data from multiple smartphone sensors and logs (Table 1) [8,26]. GPS data were collected for a 1-minute interval followed by a 10-minute interval of noncollection, that is, GPS data were collected for 1 minute every 11 minutes (hence, approximately 6 times per hour).

### Statistical Methods

#### Data Volume

For clinic-based and smartphone-based surveys, we reported the number of clinic-based and smartphone-based ALSFRS-R surveys per participant. For GPS location data, we reported the number of participant-days for which data were available and the total data volume. Data were considered available if the app had recoded any location data on that day.

#### Kaplan–Meier Estimates of Time-to-Discontinuation

For smartphone survey data, we defined the date of dropout as the date of the first missed survey, that is, the week after a participant had completed their last smartphone survey. For smartphone sensor data, we defined the date of dropout as the day after the last recording of smartphone sensor data.

We used the Kaplan–Meier method to estimate time-to-discontinuation for smartphone survey data (model 1) and for smartphone sensor data (model 2). Both models were stratified by study type. Time-to-discontinuation was censored at the end of each study’s follow-up period (after week 12, 52, or 20; see Table 1) if a participant died and, for trial participants, if they discontinued the trial because of side effects.

#### Proportional Hazard of Dropping Out

We used Cox proportional hazard regression to identify the predictors of smartphone survey data and smartphone sensor data. We tested whether the likelihood of dropout was higher for participants with certain demographic characteristics or with a higher disease severity at baseline.

The covariates we included in the model were as follows:

- Participant characteristics, such as age (in years), sex (male or female), and smartphone operating system (Android or iOS)
- Disease severity at baseline, such as baseline functional ability as measured by the 4 domains of the ALSFRS-R score. These four domains are the fine motor domain score, gross motor domain score, bulbar domain score, and respiratory domain score.

#### Data Completeness

Data completeness was defined as the percentage of days for which participants provided GPS data and the percentage of weeks for which participants submitted surveys and audio recordings. For GPS data, 100% data completeness meant that GPS data were available for each day from the participants’ enrollment until their last day in the study. For survey and audio recording data, 100% data completeness meant that a participant had submitted 1 survey or audio recording per week for each week from their enrollment until their last day in the study.

We visualized the data in a boxplot of participants’ data completeness during the time they contributed to the data. In addition, we calculated the data completeness for each 28-day period in which a participant was in the study. We used a 28-day period rather than a calendar month, because the total duration of all 3 studies was a multitude of 28 days; the maximum time in study was 3×28 days for study 1, 12×28 days for study 2, and 5×28 days for study 3. We plotted data completeness for each 28-day period (hereafter, *month*), stratified by participants’ total duration in the study.

## Results

The 3 studies are referred to as *study 1* (12-week observational pilot study), *study 2* (52-week observational study) and *study 3* (20-week clinical trial) in this section.

### Participants

Demographic data for the 3 studies are presented in Table 2. There were 22 participants in study 1, 49 in study 2, and 23 in study 3. There were more male participants in the 2 observational studies (15/22, 68% and 29/49, 59%; in line with a higher prevalence of ALS in men), but fewer in the clinical trial (9/23, 39%). The mean age and baseline ALSFRS-R scores were similar across studies. Owing to the differences in inclusion criteria, mean disease duration was 5-7 months shorter for participants in the clinical trial, and baseline mean vital capacity, a measurement of lung volume, was higher for those in the clinical trial. Most participants were iOS users in all studies (56/94, 60%). In each of the three studies, one person died before the end of the study.

In total, we collected 185 ALSFRS-R scores during clinic visits (43 for study 1, 77 for study 2, and 65 for study 3), 1465 ALSFRS-R scores from smartphones (375 for study 1, 759 for study 2, and 331 for study 3), 3748 audio recordings from smartphones (678 for study 1, 1315 for study 2, and 609 for study 3), and a total of 10.4 GB of GPS location data (3.4 GB for study 1, 5.5 for study 2, and 1.5 GB for the study 3).

**Table 2:** Demographic characteristics of participants per study.

Characteristics		Study 1	Study 2	Study 3
Number of participants, N		22	49	23
Sex (male), n (%)		15 (68)	30 (59)	9 (39)
Race (White), n (%)		20 (91)	48 (98)	23 (100)
Phone operating system (iOS users), n (%)		17 (77)	36 (73)	12 (52)
**Location of symptom onset, n (%)**		21 (100)	49 (100)	23 (100)
	Bulbar	5 (23)	11 (22)	7 (30)
	Limb	16 (73)	38 (78)	15 (65)
	Trunk	1 (5)	0	1 (4)
Age (years), mean (SD)		56 (6)	57 (11)	58 (10)
Disease duration at baseline visit (months), mean (SD)		31 (21)	35 (23; n=48)^a^	26 (14; n=22)^a^
Diagnostic delay^b^ (months), mean (SD)		17 (13)	17 (14)	12 (7; n=22)^a^
**Baseline ALSFRS-R^c^ total score, mean (SD)**		34 (7)	35 (9; n=46)^a^	36 (8)
	Bulbar subscore	10 (2)	10 (3)	9 (3)
	Fine motor subscore	8 (2)	8 (3)	8 (3)
	Gross motor subscore	7 (3)	7 (3)	8 (3)
	Respiratory subscore	9 (3)	10 (2)	11 (2)

^a^Data were missing; mean and SD calculated over smaller sample size (smaller sample size provided as n, wherever applicable).

^b^Diagnostic delay: time between symptom onset and diagnosis.

^c^ALSFRS-R: revised amyotrophic lateral sclerosis functional rating scale.

### Time-to-Discontinuation

**Figure 1:**
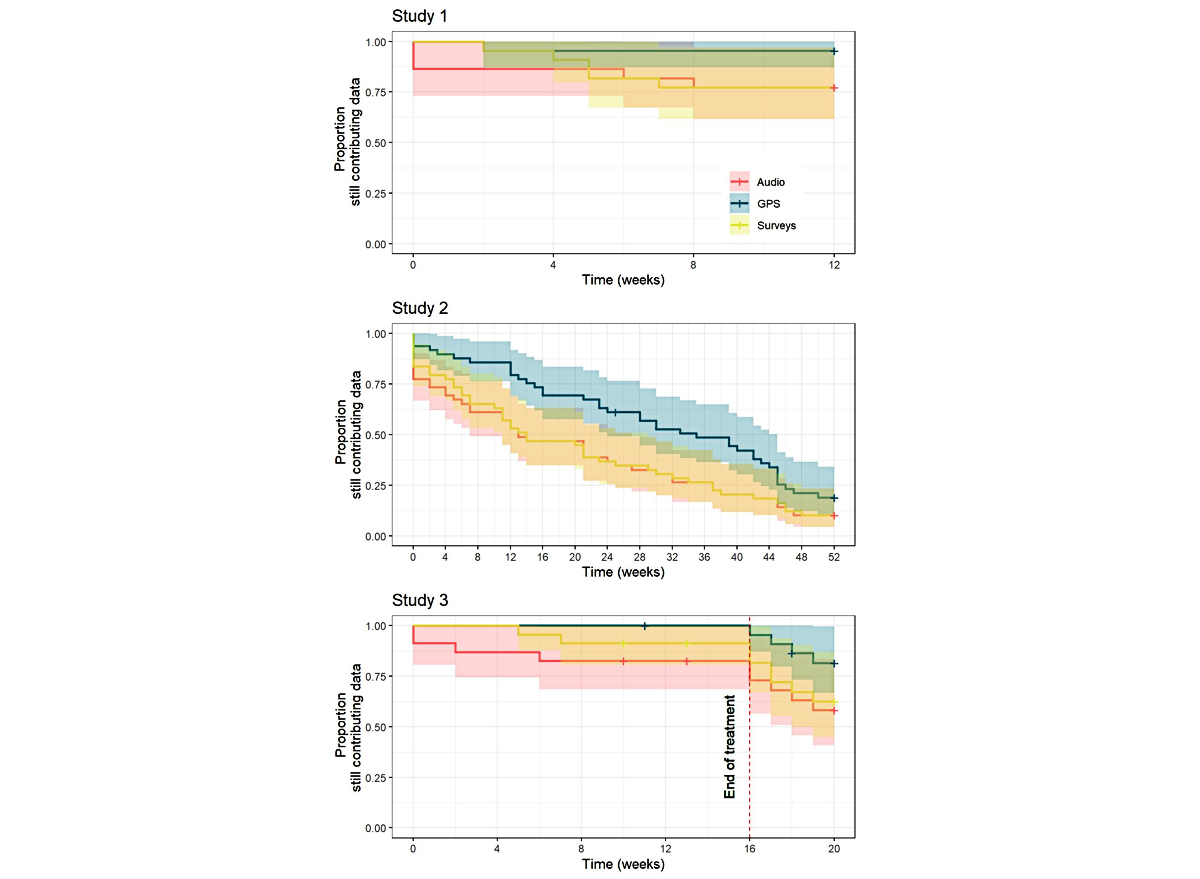
Kaplan–Meier plot estimates of time-to-discontinuation for 3 data types. Each color denotes a different data type: audio data in red, GPS data in blue, and survey data in yellow. Participants that were censored before the end of the study are denoted by + signs. Each panel shows time-to-discontinuation in a different study: study 1 (top, a 12-week pilot study), study 3 (middle, a 20-week clinical trial), and study 2 (bottom, a 1-year observational study).

Kaplan–Meier estimates of the time-to-discontinuation for active data are presented in Figure 1 (red line for audio recordings, yellow line for surveys, and blue line for passive data). After 12 weeks, 77% (17/22) of the participants in study 1, 59% (29/49) of the participants in study 2, and 96% (22/23) of the participants in study 3 continued to contribute active data (surveys and audio recordings). For passive data, 95% (21/22) of the participants in study 1, 86% (42/49) of the participants in study 2, and 100% (23/23) of the participants in study 3 continued to contribute sensor data.

### Predictors of Early Discontinuation

We used the Cox proportional hazards model to estimate whether study, participant demographics, and disease severity were associated with the risk of discontinuation, with a separate model for survey, audio recording, and GPS data. None of the variables were statistically significantly associated with the risk of discontinuation. The estimated associations between the study, participant demographics, and disease severity at baseline are presented in Multimedia Appendix 1.

### Data Completeness

The time-to-discontinuation model described above paints only part of the picture—how long the participants contributed to *any* data. We also explored data completeness, the proportion of days a participant provided GPS data, surveys, audio recordings of coughs, and audio recordings when participants recited a short text that was displayed on their screen. Data were 100% complete for GPS if any data were contributed for a given day, and data were 100% complete if surveys and audio recordings were completed each week when the task was presented.

**Figure 2:**
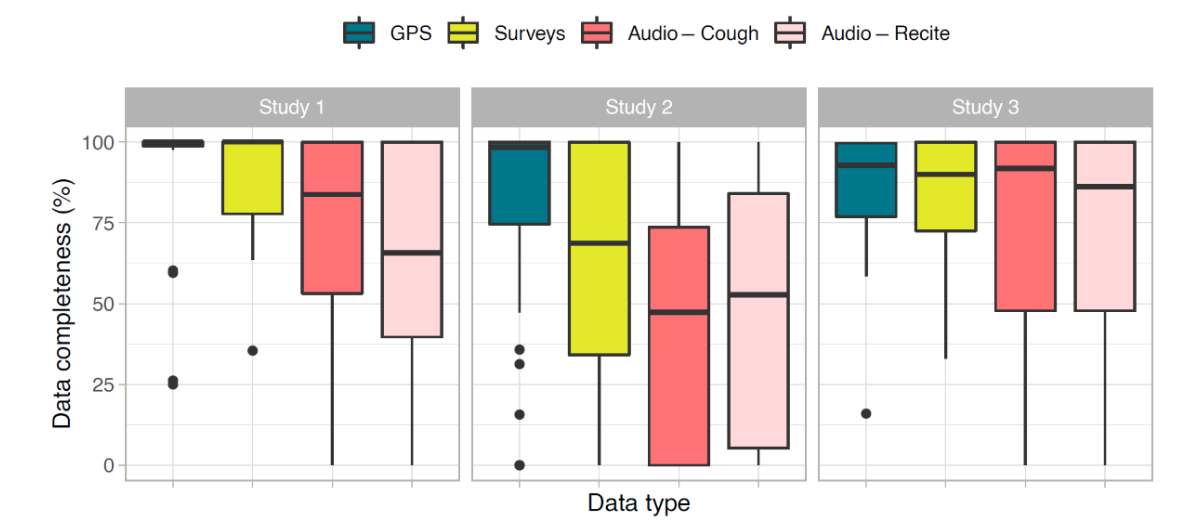
Boxplot of participants’ data completeness (in %) excluding the period after discontinuation. Data completeness was defined as percentage of days with any GPS data and percentage of weeks with a completed survey or audio recording.

Figure 2 shows boxplots for each study of the average data completeness for each data type before discontinuation (after discontinuation, data completeness is 0% by definition). In all studies, GPS data completeness was highest over the 3 studies (range 90%-100% of days that a participant stayed in the study), followed by survey data in study 1 (median 100%) and study 2 (median 90%) and by audio recordings (cough recordings; median 92%) in study 3. Of the 3 studies, study 3 (20-week clinical trial) had the highest data completeness, and study 2 (52-week observational study) had the lowest data completeness.

**Figure 3:**
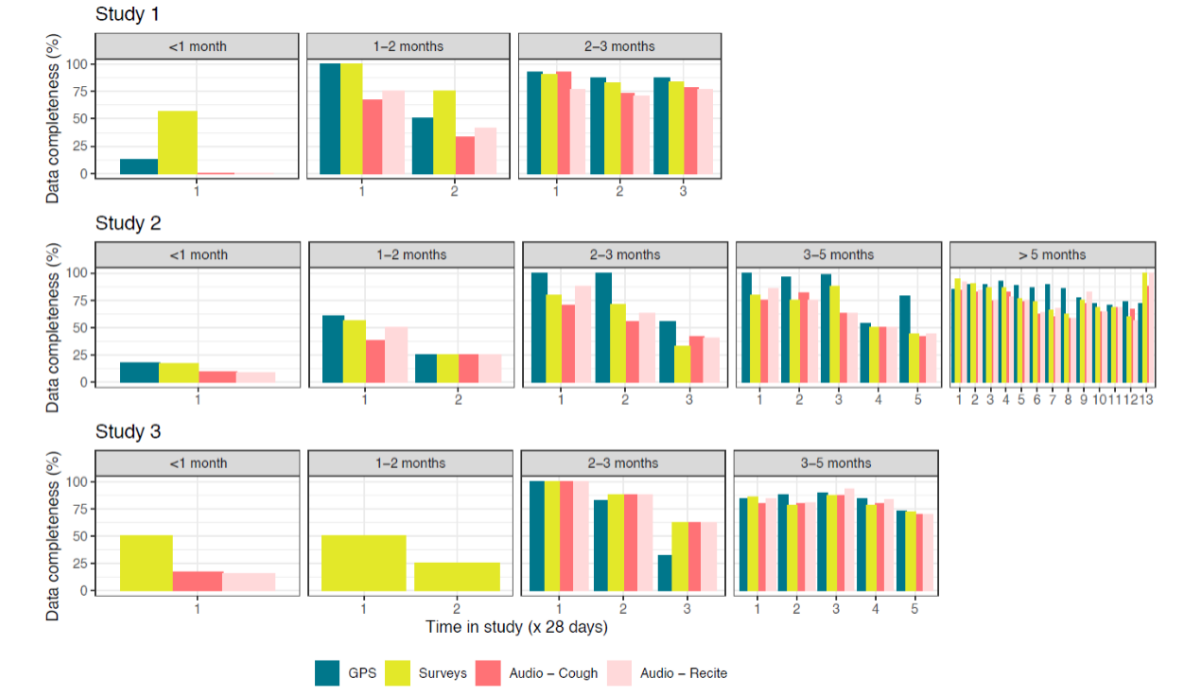
Bar graph of data completeness per month in study (excluding the period after discontinuation), stratified by time-to-discontinuation of the participant (gray bar indicates time-to-discontinuation). Number of participants for each panel from left to right are as follows: N=7, 4, and 18 for study 1; N=20, 6, 10, 8, and 33 for study 2; and N=5, 1, 2, and 22 for study 3. Data completeness was defined as percentage of days with any GPS data and percentage of weeks with a completed survey or audio recording.

We then plotted data completeness for each 28-day period (hereafter, *month*), stratified by participants’ total duration in the study (Figure 3). First, this analysis showed that participants who contributed data for longer (eg, for >2 months) had higher data completeness than participants who stopped contributing data in the first or second month. Participants who contributed data longer had a data completeness fluctuating around 75% for all the data types for their first months in the study, whereas early dropouts typically had low data completeness for the months that they were active. For those dropping out within the first month, mean data completeness across the studies ranged from 7.8% (audio recording) to 41% (surveys). For those who dropped out in the second study month, completeness ranged from 41% (audio cough recording) to 59% (GPS data). Second, for most participants, data completeness declined over time; mean data completeness was typically lower in the last month of the study (except for study 2, where participants who were active until the final month completed all surveys and audio tasks). Third, data completeness was generally highest for GPS data (except for those who dropped out in the first month). Fourth, data completeness did not differ significantly between the 2 audio tasks.

## Discussion

### Principal Findings

In this study, we showed that smartphones can be used to collect frequent active and passive data from people with neurodegenerative diseases, specifically ALS, both in observational studies and in a clinical trial setting. Participant engagement, as measured by time-to-discontinuation, was higher than that in published data [9,10]. The two observational studies described in this paper, in which no adherence reinforcement or incentives were implemented, provide an important benchmark for participant engagement with a smartphone app in research.

Data completeness was lower for active data than for passive data. In other words, smartphones continued to collect passive data even after participants had stopped completing surveys or recording audio.

**Understanding Participant Adherence**

Time-to-discontinuation was higher in our studies compared with smartphone data collection studies in other domains, which often show an exponential dropout [9,10,27]. The lower dropout rate in our study may highlight the high commitment to research of people with ALS and neurodegenerative diseases despite the challenges of progressive functional and cognitive decline. In another smartphone study, participants with multiple sclerosis dropped out significantly later than healthy controls, who only remained active for 1 day [19]. In addition, time-to-discontinuation was shorter for clinic-referred participants than for self-referred participants (7 days vs 25.5 days) and shorter than in our study.

Our analysis did not provide evidence that demographic characteristics or disease severity at baseline were associated with attrition, although our analysis was underpowered to detect predictors of attrition.

### Strengths and Limitations

ALS is a rare disease, and our analysis of 3 studies, both in observational and interventional research contexts, is the first of its kind. Given that sample sizes were limited by the low prevalence of ALS, we were underpowered to detect associations between participant and disease characteristics and adherence to digital data collection. Furthermore, although neurodegenerative diseases share many characteristics, our results may not be generalizable to all neurodegenerative diseases.

### Improving Participant Adherence

Despite better than expected adherence compared with published studies, boosting adherence remains important, especially for clinical trials using smartphone-based outcomes. Participants of digital health studies are more likely to actively engage long term if they see the value of participation [14,27], which may have been the case, especially in the trial participants who received a novel therapeutic. Personal contact with study personnel helps participants feel valued and is a major driver of engagement [27]. Both perceived value and personal contact with study personnel may explain the better participant adherence in study 3, which had almost full adherence until the end of treatment with the study drug.

In future studies, we will test whether reminder phone calls, more frequent clinic visits, or financial incentives can improve adherence, particularly in longer studies. Another potential motivator for adherence could be allowing participants to view their data, including previous survey responses [28]. However, this may not always be scientifically advisable, as it may influence participants’ responses through the Hawthorne effect and related forms of reporting bias [29].

Data completeness was higher and attrition was lower for passive data than for active data. Passive data incompleteness is due to both behavioral factors (eg, a participant disabling GPS) or technological issues (eg, smartphone blocking sensor data collection) [12,30,31]. Investigators familiar with passive smartphone data collection recognize that both commonly used smartphone operating systems (Android and iOS) implement power saving measures for apps running in the background to reduce consumption of the central processing unit resources, memory, and battery [12,31]. This means that no app can run in the background mode indefinitely, but instead the app needs to be brought to the foreground at least occasionally for the background data collection to persist [30]. Therefore, longitudinal passive data collection without active data collection is not possible. Factors such as device type, hardware, and operating system influence data completeness [30]. These technological factors can be difficult to modify, and they also change over time.

### Identifying High Adherence: Run-in and Withdrawal Design

Our analyses showed that participants in the clinical trial adhered best to the study regimen. When treatment ended, >80% were still answering surveys, and all eligible participants were still contributing sensor data. Nevertheless, for clinical trials, it could be useful to identify participants who are more likely to adhere. For studies requiring participants to use smartphones, especially trials, a *run-in and withdrawal design* has been suggested [9]. With this design, participants enter a *weed-out* period after enrollment. Only participants who still used the study app after the weed-out period were randomized. Our study showed that participants who stopped contributing surveys within 1 or 2 months of enrolling had lower data completeness than their engaged counterparts. This suggests that monitoring active data completeness during a screening period for a trial could help identify participants who are more likely to adhere.

### Conclusions

Our study demonstrates that it is possible to collect longitudinal research data from people with progressive neurodegenerative diseases using their personal smartphones. Our results are especially promising for clinical trials (longer time-to-discontinuation than in observational studies) and for studies collecting mainly passive data with a light active data component (higher data completeness and longer time-to-discontinuation than in studies prioritizing survey data). We identified putative predictors of dropout, which can be confirmed in future studies, and will allow researchers to target efforts to improve participant adherence to smartphone data collection.

## Appendix

Multimedia Appendix 1
Results of Cox models.

## Abbreviations

ALS: amyotrophic lateral sclerosis
ALSFRS-R: revised amyotrophic lateral sclerosis functional rating scale
IRB: Institutional Review Board
MGH: Massachusetts General Hospital
