# Supplementary material for: Using Smartphones to Reduce Research Burden in a Neurodegenerative Population and Assessing Participant Adherence: A Randomized Clinical Trial and Two Observational Studies
Source: JMIR Mhealth Uhealth. 2022 Feb 4;10(2):e31877. doi: 10.2196/31877 (PMC8857693; doi:10.2196/31877)
Supplement: Multimedia Appendix 1 [file mhealth_v10i2e31877_app1.docx]

Supplementary materials

Multimedia Appendix 1. Results of Cox models

| SURVEY DATA |  |  |  |
| --- | --- | --- | --- |
|  | **Risk Ratio** | **95% Confidence Interval** | **P value** |
| Study 2 (ref: Study 1) | 2.51 | (0.9 - 6.99) | 0.078 |
| Study 3 (ref: Study 1) | 1.3 | (0.38 - 4.38) | 0.677 |
| Age (per year) | 0.98 | (0.95 - 1.01) | 0.247 |
| Male sex (ref: female) | 1.39 | (0.77 - 2.52) | 0.278 |
| Operating system iOS (ref: Android) | 1.08 | (0.56 - 2.06) | 0.825 |
| ALS-FRSR domain score |  |  |  |
| Bulbar domain (per point) | 1 | (0.9 - 1.11) | 0.943 |
| Small motor domain (per point) | 0.99 | (0.86 - 1.14) | 0.913 |
| Gross motor domain (per point) | 0.98 | (0.88 - 1.1) | 0.784 |
| Respiratory domain (per point) | 0.92 | (0.81 - 1.05) | 0.234 |

| AUDIO SURVEY DATA | |  |  |
| --- | --- | --- | --- |
|  | **Risk Ratio** | **95% Confidence Interval** | **P value** |
| Study 2 (ref: Study 1) | 2.76 | (0.99 - 7.69) | 0.051 |
| Study 3 (ref: Study 1) | 1.75 | (0.53 - 5.81) | 0.357 |
| Age (per year) | 0.98 | (0.95 - 1.01) | 0.224 |
| Male sex (ref: female) | 1.31 | (0.73 - 2.35) | 0.363 |
| Operating system iOS (ref: Android) | 1.26 | (0.65 - 2.45) | 0.495 |
| ALS-FRSR domain score |  |  |  |
| Bulbar domain (per point) | 0.97 | (0.87 - 1.07) | 0.51 |
| Small motor domain (per point) | 1.01 | (0.88 - 1.16) | 0.895 |
| Gross motor domain (per point) | 0.99 | (0.88 - 1.11) | 0.847 |
| Respiratory domain (per point) | 0.92 | (0.81 - 1.05) | 0.215 |

| GPS DATA |  |  |  |
| --- | --- | --- | --- |
|  | **Risk Ratio** | **95% Confidence Interval** | **P value** |
| Study 2 (ref: Study 1) | 4.76 | (0.55 - 40.98) | 0.155 |
| Study 3 (ref: Study 1) | 2.5 | (0.24 - 25.61) | 0.44 |
| Age (per year) | 1 | (0.96 - 1.03) | 0.885 |
| Male sex (ref: female) | 0.7 | (0.35 - 1.38) | 0.299 |
| Operating system iOS (ref: Android) | 1.72 | (0.84 - 3.52) | 0.135 |
| ALS-FRSR domain score |  |  |  |
| Bulbar domain (per point) | 1.13 | (0.98 - 1.3) | 0.098 |
| Small motor domain (per point) | 1.04 | (0.86 - 1.24) | 0.697 |
| Gross motor domain (per point) | 1.06 | (0.93 - 1.22) | 0.383 |
| Respiratory domain (per point) | 0.86 | (0.71 - 1.03) | 0.093 |
| Still completing surveys (ref: disengaged | 0.91 | (0.43 - 1.92) | 0.807 |
